# A Cohort Study on the Comparison of Complications, Short-Term Efficacy, and Quality of Life between Thoracoscopic Surgery and Traditional Surgery in the Treatment of Rib Fractures

**DOI:** 10.1155/2022/2079098

**Published:** 2022-05-18

**Authors:** Dongdong Wang, Yongdong Xu, Qingqing Wang, Yueping Xu, Xiaoqi Wang

**Affiliations:** Department of Thoracic Surgery, Shanghai Pudong Hospital Fudan University Pudong Medical Center, Shanghai 201399, China

## Abstract

**Objective:**

A case-control study was conducted, to assess the complications, short-term effectiveness, and quality of life of video-assisted thoracoscopic surgery with conventional surgery in the treatment of rib fractures.

**Methods:**

From February 2018 to April 2021, 100 patients with rib fractures who required surgical treatment at the hospital were selected. Patients were randomly divided into control and study groups. The study group received thoracoscopy-assisted rib internal fixation, and the control group received traditional open reduction and internal fixation for rib fractures. The treatment effect, postoperative complication rate, surgery-related indicators, stress response, blood gas indicators, VAS (visual analog scale) pain score, and SF-36 quality of life score were compared between the two groups.

**Results:**

The total effective rate of the study group was higher than that of the control group, and the difference was statistically significant (*P* < 0.05). The postoperative complications in the study group were significantly lower than those in the control group (*χ*^2^-5.317; *P* < 0.05), but there was no significant difference in hospitalization costs between the two groups (*P* > 0.05). The operation time, intraoperative blood loss, incision length, drainage tube placement time, postoperative activity time, and hospital stay in the study group were significantly lower than those in the control group. The SF-36 score and VAS score in the study group were higher than those in the control group (*P* < 0.05). Compared with the two groups after the operation, the levels of PaO2, SaO2, and PaO2/FiO2 in the study group were significantly higher than those in the control group (*P* < 0.05). Before surgery, there was no significant difference in stress response indicators such as cortisol, blood sugar, and C-reactive protein between the two groups (*P* > 0.05), but there was no significant difference in stress response indicators after surgery (*P* > 0.05). Cortisol, blood sugar, C-reactive protein, and other indicators were increased in both groups, but compared with the control group, the study group had decreased postoperative cortisol, blood sugar, C-reactive protein, and other stress response indicators (*P* < 0.05).

**Conclusion:**

There is a significant difference between thoracoscopic surgery and traditional surgery in the treatment of rib fractures. The probability of postoperative complications of thoracoscopic surgery is lower, and the operation time, intraoperative blood loss, and incision length are better. The pain of patients before and after the operation is significantly reduced, the quality of life is improved greatly, and the stress response is weak.

## 1. Introduction

In recent years, because of the rapid economic development, the number of patients with chest trauma induced by traffic, engineering accidents, and mining accidents has gradually increased. In chest trauma, rib fracture is the most common, which is a common disease in thoracic surgery. In particular, multiple rib fractures caused by severe chest trauma are often induced by flail chest, lung contusion and laceration, and hemopneumothorax, resulting in a complex injury, critical condition, and respiratory failure or circulatory failure [1–3]. A series of chest pathological changes induced by rib fracture cannot be ignored: (1) the broken end of the fracture stimulates the intercostal nerve to cause chest pain, resulting in shallow breathing and weak cough, which can easily lead to atelectasis and pulmonary infection; (2) the broken end of the fracture can pierce the pleura, intercostal vessels, and lung tissue, resulting in hemothorax, pneumothorax, subcutaneous emphysema, or hemoptysis; (3) multiple rib fractures softened the local chest wall without complete rib support, resulting in abnormal respiratory movement, affecting the pulmonary ventilation and blood circulation, and causing respiratory and circulatory failure in severe cases; (4) large area pulmonary interstitial and alveolar edema induced by large area pulmonary interstitial and alveolar edema. Meanwhile, alveolar cell injury promoted diffuse dysfunction, leading to hypoxemia and even acute respiratory distress syndrome [4, 5]. At present, the treatment of multiple rib fractures is mainly divided into conservative treatment and surgical treatment. The most important procedure in the treatment is to maintain the stability of the chest wall, reduce the degree of chest pain, and return to normal breathing. Conservative treatment often uses chest bandage, rib traction, ventilator-assisted breathing, and so on, but nonoperative treatment has some disadvantages, such as many complications, long treatment time, obvious pain, and slow healing [2]. With the development of surgical materials and thoracic surgeons' understanding of the disease, internal fixation of rib fractures as soon as possible to eliminate a series of problems induced by thoracic instability has been gradually accepted, and the advantages of surgical treatment have become increasingly prominent. A large amount of data also shows that surgical reduction and internal fixation have obvious advantages over conservative treatment on the premise of ensuring the stability of vital signs. In recent years, with the deepening of the concept of minimally invasive surgery and the continuous development of video-assisted thoracoscopic surgery [6], internal rib fixation assisted by video-assisted thoracoscopy has been reported at home and abroad. The application of video-assisted thoracoscopy in the diagnosis and treatment of multiple rib fractures has been gradually recognized by experts at home and abroad. The application of video-assisted thoracoscopy in internal fixation of rib fracture is helpful to accurately evaluate the fracture site and provide accurate guidance for the selection of surgical incision, which has obvious advantages in intrathoracic exploration and injury management [7–9]. Based on this, the purpose of this study is to analyze the difference between thoracoscopic surgery and traditional surgery in the treatment of rib fractures.

To do so, the rest of the article is organized as follows. In Section 2, the patients' information, treatment methods, and evaluation index are investigated. In Section 3, the statistical analysis using the SPSS software is given. In Section 4, the results and comparisons are discussed. In Section 5, the discussion and conclusion are given.

## 2. Patients and Methods

### 2.1. Patients' Information

One hundred patients with severe sepsis were selected from February 2018 to April 2021. The patients were randomly divided into control group and study group. The age of the control group was 43–74 years, with an average of 65.91 ± 3.63 years, including 28 males and 22 females; the study group was 44–76 years old, with an average of 65.96 ± 3.58 years, including 26 males and 24 females. There was no statistical significance in the general data of the two groups. This study was approved by the Medical Ethics Association of the hospital, and all patients signed informed consent.

The inclusion criteria were as follows: (1) the age was ≥18 years old; (2) the patients had good communication skills and no language barrier and could actively cooperate with the relevant scores, examinations, and inquiries; (3) the vital signs were stable and there was no need for emergency operation; (4) there were no obvious surgical taboos in arterial blood gas analysis and preoperative tests.

The exclusion criteria were as follows: (1) patients with severe heart, liver, renal insufficiency, malignant tumors, and other diseases; (2) patients with long-term infection or recent infection not cured after treatment, or infection has been cured for less than one year; (3) patients with serious compound injuries such as head, abdomen, spine, limbs, and so on.

### 2.2. Treatment Methods

The patients in the control group were treated with traditional operation. According to the three-dimensional reconstruction of chest CT and ribs, combined with body surface compression, at the center of the broken and gathered area of chest ribs on the affected side, the 10∼15 cm incision was taken along the shape of the ribs, the skin, subcutaneous tissue, and muscles were cut layer by layer, the fracture lines were fully exposed, appropriate intercostals were selected, intercostal muscles were cut, and rib retractors were used to stretch into the chest to complete hemothorax cleaning, hemostasis, repair of lung rupture, and so on. The ribs with obvious dislocation and supporting function were selected and fixed with Watson rib plate of suitable specification. At the end of the operation, a closed thoracic drainage tube was placed to complete the operation. The study group was treated with thoracoscopic-assisted rib internal fixation, and the 7^th^ intercostal or thoracic closed drainage hole in the axillary midline of the affected side was selected as the observation hole, and the specific conditions of intrathoracic and rib fractures were observed by video-assisted thoracoscopy. Under the guidance of video-assisted thoracoscopy, combined with body surface compression, obvious dislocation or broken ribs of the pleura were located, and the central part of the rib fracture that needed to be fixed was selected to make a 4 cm incision along the ribs to bluntly separate the chest wall and intercostal muscle. From this fracture, the operation hole was made between the ribs, and the incision protective cover was placed to complete the cleaning of hemothorax, hemostasis, repair of lung rupture, and so on. The fractured end was exposed by blunt separation, the periosteum was not peeled off, the dislocated ribs were dissected and reduced, and the Watson rib plate with suitable specifications was selected to complete the fixation. According to the principle of “key fixation,” only the main supporting ribs are fixed, and the broken ribs which are adjacent, not seriously fractured, and not obviously dislocated are not fixed. The rest are far away from the incision, and the fixed ribs need to be fixed with the same small incision method. Under direct vision, sputum suction and lung bulging were performed, and the closed thoracic drainage tube was placed through the observation hole to complete the operation.

### 2.3. Evaluation Index

#### 2.3.1. Evaluation of Curative Effect and Postoperative Complications

1. The patients were followed up for six months after discharge, and the overall evaluation was made according to the postoperative curative effect index. Effective: slight abnormal sensation and pain in the chest wall, no movement and respiratory dysfunction; effective: abnormal sensation and pain in the chest wall, dissatisfaction with the appearance of the chest wall, mild movement, and respiratory dysfunction, does not affect life and work; invalid: left behind chest wall sensory abnormality and pain, dissatisfied with the appearance of the chest wall, movement, and respiratory dysfunction, affecting life and work. Efficiency = (effective + effective)/number of cases ^*∗*^ 100%.
2. The postoperative complications of pulmonary infection, atelectasis, incision infection, and pleural effusion in the two groups were recorded.

#### 2.3.2. Comparison of Operation-Related Indexes between the Two Groups

The time of operation, the amount of blood loss during operation, the length of surgical incision, the placement time of drainage tube, the time of getting out of bed after operation, the time of hospitalization, and the cost of hospitalization were included. Postoperative complications included pulmonary infection, atelectasis, pleural effusion, and incision infection.

#### 2.3.3. Comparison of Stress Response Indexes between the Two Groups

Fasting blood samples were collected on the first day before operation and the first day after operation, and the levels of serum cortisol, blood glucose, and C-reactive protein were measured. C-reactive protein was determined by latex enhanced scatter immunoturbidimetry and Abbott C8000 automatic biochemical instrument.

#### 2.3.4. Blood Gas Examination Method

Arterial blood samples were taken to detect blood gas analysis, and partial pressure of arterial oxygen (PaO2), partial pressure of arterial blood carbon dioxide (PaCO2), arterial oxygen saturation (SaO2), and oxygenation index (PaO2/FiO2) were recorded. Oxygenation index is an important index to evaluate the respiratory function of patients. When oxygenation index is lower than 200, patients have acute lung injury. If oxygenation index is lower than 150, patients have acute respiratory distress syndrome.

#### 2.3.5. Pain Score and Quality of Life Evaluation Method

Visual analogue scale (VAS) [9] was used to score the pain of the patients before and after treatment: draw a straight line on the paper, divided into 10 equal parts, marked 0–10 in turn to indicate that the pain was normal, 10 indicated that the pain was severe and unbearable, and the middle part indicated that the pain degree increased with the increase of the value.

The SF-36 health survey scale [10] was used to analyze the quality of life of the two groups after nursing, with eight dimensions including emotional function, social function, and physical pain, a total of 36 items. Each item scored 0–6 points, and the average score was taken. The higher the score, the better the patient's quality of life.

## 3. Statistical Analysis

The professional statistical software SPSS21.0 was used for data processing and analysis. The metrological data were expressed by mean soil standard deviation as $\bar{x}$ ±*s*, the comparison between groups was expressed by *t*-test, and the counting data was expressed by rate *n* (%). Χ^2^ test was used for comparison between groups, when *P* < 0.05 the difference was statistically significant.

## 4. Results and Comparisons

### 4.1. Comparison of the Therapeutic Effects

We followed up all patients, and none of the patients relapsed during the follow-up period. First, we compared the metabolism of the two groups. In the study group, 27 cases were markedly effective, 22 cases were effective, and one case was ineffective, and the total effective rate was 98.00%. In the control group, 15 cases were markedly effective, 28 cases were effective, and 13 cases were ineffective. The total effective rate of treatment was 86.00%. The total effective rate of the study group was higher than that of the control group, and the difference was statistically significant (*P* < 0.05). All the data results are shown in Table 1.

### 4.2. Comparison of the Probability of Postoperative Complications

The main postoperative complications of the two groups were pulmonary infection, atelectasis, incision infection, and pleural effusion. There were one case of atelectasis, one case of incision infection, and one case of pleural effusion in the study group, and the probability of postoperative complications was 6.00%. In the control group, there were two cases of pulmonary infection, three cases of atelectasis, two cases of incision infection, and four cases of pleural effusion. The probability of postoperative complications was 22.00%. Compared with the control group, the postoperative complications in the study group were significantly lower than those in the control group, and the difference was statistically significant (*χ*^2^ = 5.317 *P* < 0.05).

### 4.3. Comparison of the Operation-Related Indexes between the Two Groups

We compared the operation-related indexes between the two groups, and there was no significant difference in hospitalization costs between the two groups (*P* > 0.05). The operation time, intraoperative blood loss, incision length, drainage tube placement time, postoperative activity time, and hospitalization time in the study group were significantly lower than those in the control group (*P* < 0.05). All the results are shown in Table 2.

### 4.4. Comparison of SF-36 Score and VAS Score between the Two Groups before and after the Operation

We compared the SF-36 score and VAS score between the two groups before and after operation. There was no significant difference in SF-36 score and VAS score between the two groups before operation. Compared with those before operation, the SF-36 score of the two groups increased and the VAS score decreased. Compared with the control group, the SF-36 score of the study group was significantly higher and the VAS score was lower, and the difference was statistically significant. All the data are shown in Table 3.

### 4.5. Comparison of Blood Gas Analysis between the Two Groups before and after the Operation

We compared the blood gas analysis between the two groups before and after the operation. There was no significant difference in the levels of PaO2, PaCO2, SaO2, and PaO2/FiO2 between the two groups before operation. After operation, the PaCO2 of the two groups decreased and the levels of PaO2, SaO2, and PaO2/FiO2 increased. Compared between the two groups, the level of PaCO2 in the study group after operation was lower than that in the control group, while the levels of PaO2, SaO2 and PaO2/FiO2 in the study group were higher than those in the control group, and the differences were statistically significant. All the data are shown in Table 4.

### 4.6. Comparison of Stress Response Indexes between the Two Groups before and after the Operation

The stress response indexes of the two groups before and after the operation were compared. There was no significant difference in cortisol, blood glucose, and C-reactive protein between the two groups before the operation (*P* > 0.05). After the operation, the stress response indexes such as cortisol, blood glucose, and C-reactive protein in the two groups were increased, but compared with the control group, the stress response indexes such as cortisol, blood glucose, and C-reactive protein in the study group were lower than those in the control group. The differences were statistically significant (*P* < 0.05). All the data are shown in Table 5.

## 5. Discussion

Rib fractures are one of the more common chest injuries [11–13]. Rib fractures are found in 40% to 60% of chest injuries. Direct or indirect violence can lead to rib fractures. The focus point of the chest wall is prone to be subjected to direct action and lead to fracture, which often leads to the fracture of the fourth to eighth ribs, which can also promote internal organ damage, including nerves and blood vessels. Blood vessels can also be indirectly damaged [14]. At present, the treatment of rib fracture is mainly divided into conservative treatment and surgical treatment. The most important thing in the treatment is to maintain the stability of the chest wall, reduce the degree of chest pain, and return to normal breathing [6]. Conservative treatment often uses chest band pressure bandaging, rib traction, ventilator-assisted breathing, and so on, but nonoperative treatment has many complications, long treatment time, obvious pain, slow healing, and other shortcomings [15]. With the development of surgical materials and thoracic surgeons' understanding of the disease, internal fixation of rib fractures as soon as possible to eliminate a series of problems caused by thoracic instability has been gradually accepted, and the advantages of surgical treatment have become increasingly prominent [16]. A large amount of data also shows that, under the premise of ensuring the stability of vital signs, surgical reduction and internal fixation has obvious advantages over conservative treatment. At present, internal fixation is advocated for the treatment of rib fracture including flail chest. The broken end of rib fracture was fixed by operation to ensure the good alignment of the broken end and the integrity and stability of the chest, effectively eliminate the harmful effect of the movement of the broken end of rib fracture on the surrounding tissue, and promote the reexpansion of lung tissue as soon as possible. Lung ventilation is improved, and therefore, the patients' quality of life is improved [17]. Studies have shown that early internal fixation can improve the environment of fracture healing and reduce the occurrence of thoracic deformities, chronic pain, and lung diseases induced by nonunion or malunion [18]. As most of the patients with rib fracture are complicated with lung contusion and laceration, flail chest, hemopneumothorax, and other intrathoracic organ injuries, intrathoracic exploration, hemostasis, and repair of injuries should be performed at the same time of rib internal fixation [19–21]. The traditional open reduction and internal fixation of rib fracture combined with thoracotomy exploration has obvious disadvantages: large incision, large injury, long postoperative recovery time, and high incidence of postoperative complications [22]. With the development and progress of endoscopic technology, video-assisted thoracoscopy technology has become increasingly mature, and clinicians have gradually mastered the technology of video-assisted thoracoscopy. Video-assisted thoracoscopy has been more and more widely used in the emergency treatment of chest trauma [23, 24].

In this study, the total effective rate of the study group was 98.00%, while that of the control group was 15 cases, 28 cases, and 13 cases, respectively, and the total effective rate of the study group was 86.00%. The total effective rate of the study group was higher than that of the control group. The total effective rate of the study group was higher than that of the control group; the total effective rate of the study group was higher than that of the control group. The total effective rate of the study group was significantly higher than that of the control group. The main postoperative complications of the two groups were pulmonary infection, atelectasis, incision infection, and pleural effusion. There was one case of atelectasis, one case of incision infection, and one case of pleural effusion in the study group, and the probability of postoperative complications was 6.00%. In the control group, there were two cases of pulmonary infection, three cases of atelectasis, two cases of incision infection, and four cases of pleural effusion. The probability of postoperative complications was 22.00%. Compared with the control group, the postoperative complications in the study group were significantly lower than those in the control group, and the difference was statistically significant (*χ*^2^ = 5.317 *P* < 0.05). The operation time, intraoperative blood loss, incision length, drainage tube placement time, out-of-bed movement time, hospital stay time, operation time, and intraoperative blood loss in the study group were 135.33 ± 24.34 min, 115.38 ± 33.51 ml, 9.23 ± 3.53 cm, 73.54 ± 16.58 *h*, 3.38 ± 1.66 *d*, 10.38 ± 2.52 *d*, and 152.14 ± 27.43 min, and 190.98 ± 45.61 ml, respectively. The length of incision was 15.84 ± 3.42 cm, the placement time of drainage tube was 86.58 ± 17.61 *h*, the time of getting out of bed after operation was 4.88 ± 1.87 *d*, and the time of hospitalization was 14.98 ± 3.74 *d*. There was no significant difference in hospitalization expenses between the two groups (*P* > 0.05). The analysis shows that video-assisted thoracoscopy is helpful to accurately locate the fracture site, reduce the blindness of incision selection, shorten the length of incision, and shorten the operation time, while the traditional surgical localization method is not accurate. During the operation, it is inevitable to prolong the surgical incision and increase the amount of blood loss and surgical trauma. On the other hand, compared with the traditional operation group, the video-assisted thoracoscopy group did not damage the chest wall and intercostal muscles, reduced nerve and vascular collateral injury, significantly reduced intraoperative bleeding, and avoided long-term postoperative pain and dysfunction. In addition, in the traditional operation group, the chest wall incision is large, and the muscle and parietal pleural injuries are more, while in the video-assisted thoracoscopy group, the operation field is clear, the thoracoscopic-assisted cleaning of the pleural cavity is more thorough, the pleural effusion is less, and the placement time of the thoracic drainage tube is significantly shortened. Thoracoscope-assisted technique is used in internal fixation of rib fracture. The application of endoscopic instruments during the operation leads to an increase in cost, but it can accelerate the recovery of patients and shorten the time of hospitalization. It will not increase the financial burden of patients and has the advantages of less trauma, less pain, and quick recovery after operation. In this study, there was no significant difference in SF-36 score and VAS score between the two groups before operation. After operation, the SF-36 score of the two groups increased and the VAS score decreased. The SF-36 score and VAS score of the study group were higher than the corresponding SF-36 score of the control group. The reasons were as follows: in video-assisted thoracoscopic surgery, the incision on the chest wall was shorter and the epidermal nerve was cut off less; in the video-assisted thoracoscopic group, the muscle was bluntly separated to avoid large area injury of the chest wall muscle; the incision protective cover was used in video-assisted thoracoscopic surgery without chest opener to open the ribs, the injury to the intercostal nerve was small, and the postoperative pain of the patients was significantly relieved. Meanwhile, the operation under this mode has positive significance for the postoperative recovery of patients and can improve the quality of life of patients.

Some studies have shown that cortisol secretion is at its peak and stable on the first day after operation [25]. As an index of stress metabolic response, blood glucose tended to be stable 24 hours after operation with cortisol secretion. Therefore, the stress status of operation can be evaluated by cortisol, blood glucose, and other stress indexes. Inflammatory reaction is another form of stress reaction [25]. C-reactive protein is a kind of inflammatory response factor, and its expression can be significantly increased in a short time after tissue injury, usually at three days after infection. In order to avoid postoperative infection affecting the test results, we can choose to detect on the first day after operation. Combined with the results of this study, there was no significant difference in the levels of PaO2, PaCO2, SaO2, and PaO2/FiO2 between the two groups before operation, but the levels of PaCO2 decreased and the levels of PaO2, SaO2, and PaO2/FiO2 increased in the two groups after operation. The postoperative PaCO2 level in the study group was 34.41 ± 2.80 mmHg, which was lower than that in the control group 37.20 ± 2.57 mmHg. The levels of PaO2, SaO2, and PaO2/FiO2 were 96.82 ± 10.29 s, 98.51 ± 6.44 s, and 342.72 ± 26.10 s, respectively, which were significantly higher than those in the control group. The levels of PaO2, SaO2, and PaO2/FiO2 in the control group were significantly higher than those in the control group. In addition, there was no significant difference in stress response indexes such as cortisol, blood glucose, and C-reactive protein between the two groups before operation (*P* > 0.05). After operation, the stress response indexes such as cortisol, blood glucose, and C-reactive protein in the two groups increased. Compared with the control group, the stress response indexes such as cortisol, blood glucose, and C-reactive protein in the study group were 111.82 ± 16.29; 7.72 ± 1.10; 53.37 ± 11.85 ng/ml, respectively. All of them were significantly lower than those of the control group. The analysis shows that the stress response is a kind of nonspecific response after the body is stimulated by external trauma and various factors. Surgery can cause different degrees of stress response, depending on the severity of the trauma. When surgical trauma occurs, the hypothalamus-pituitary-adrenal cortex axis and sympathetic-adrenal medulla system are activated, secreting a large amount of cortisol, causing fat mobilization and insulin resistance, and increasing blood sugar. Cortisol is an important substance to maintain the stability of the environment in the body, and its plasma content is positively correlated with the duration and intensity of stimulation. In the traditional operation group, the main reason is slightly related to thoracoscopic trauma. Thoracoscope-assisted rib internal fixation has the advantages of accurate location, wide field of vision, and fine operation, which can reduce the damage to normal tissue, which shows that the application of video-assisted thoracoscopy in internal fixation of rib fracture can reduce stress reaction, reduce surgical trauma, and better protect physiological function, which is in line with the concept of rapid rehabilitation surgery. In addition, the arterial blood gas indexes of the two groups were significantly improved after operation, indicating that the pulmonary function recovered after internal fixation. The improvement of lung function in thoracoscopy group is more obvious, which also benefits from its advantages of less trauma and rapid recovery.

Taken together, there is a significant difference between thoracoscopic surgery and traditional surgery in the treatment of rib fractures. The probability of postoperative complications of thoracoscopic surgery is lower, and the operative indexes such as operation time, intraoperative blood loss and incision length are better. Furthermore, the pain of patients before and after the operation is significantly reduced, the quality of life is improved greatly, and the stress response is weak.

## 6. Conclusion

There is a significant difference between thoracoscopic surgery and traditional surgery in the treatment of rib fractures. The probability of postoperative complications of thoracoscopic surgery is lower, and the operation time, intraoperative blood loss, and incision length are better. The pain of patients before and after the operation is significantly reduced, the quality of life is improved greatly, and the stress response is weak [26–30].

## Figures and Tables

**Table 1:** Comparison of therapeutic effects between the two groups (*n* (%)).

Group	Cases	Significant effect (*n* (%))	Effective (*n* (%))		Invalid (*n* (%))	Total efficiency (*n* (%))
Control group	50	15 (30.00)	28 (56.00)	7 (14.00)	43 (86.00)
Research group	50	27 (54.00)	22 (44.00)	1 (2.00)	49 (98.00)
*χ* ^2^						4.891
*P*						0.026

**Table 2:** Comparison of operation-related indexes between the two groups ($\bar{x}$ ±s).

Group	Operation time (min)	Intraoperative bleeding volume (ml)	Notch length (cm)	Drainage tube placement (h)	Time to get out of bed after operation (d)	Hospitalization time (d)	Hospitalization expenses
Control group	152.14 ± 27.43	190.98 ± 45.61	15.84 ± 3.42	86.58 ± 17.61	4.88 ± 1.87	14.98 ± 3.74	5.18 ± 1.28
Research group	135.33 ± 24.34	115.38 ± 33.51	9.23 ± 3.53	73.54 ± 16.58	3.38 ± 1.66	10.38 ± 2.52	4.88 ± 1.31
*t* value	3.241	9.445	9.510	3.812	3.238	4.242	1.252
*P* value	0.002	0.001	0.001	0.001	0.001	0.001	0.214

**Table 3:** Comparison of SF-36 score and VAS score between the two groups before and after the operation ($\bar{x}$  ± *s*, *n* = 50).

Group	SF-36 scoring		VAS scoring	
	Before operation	After operation	Before operation	After operation
Control group	75.60 ± 3.31	78.24 ± 2.75a	7.46 ± 1.31	5.58 ± 1.11a
Research group	75.47 ± 3.29	83.17 ± 2.60b	7.50 ± 1.42	4.37 ± 0.85b
T value	0.197	9.211	0.146	6.120
*P* value	0.844	0.001	0.884	0.001

The control group before and after treatment, *aP* < 0.05; the study group before and after treatment, *bP* < 0.05.

**Table 4:** Comparison of blood gas analysis between the two groups before and after operation ($\bar{x}$  ± *s*, *n* = 50).

Group	PaO_2_ (mmHg)		PaCO_2_ (mmHg)		SaO_2_ (%)		PaO_2_/FiO_2_	
	Before operation	After operation	Before operation	After operation	Before operation	After operation	Before operation	After operation
Control group	72.41 ± 12.05	91.44 ± 10.30a	41.38 ± 3.42	37.20 ± 2.57a	80.72 ± 7.43	93.43 ± 6.53a	206.55 ± 22.58	313.55 ± 25.37a
Research group	72.38 ± 12.11	96.82 ± 10.29 b	41.29 ± 3.55	34.41 ± 2.80 b	80.69 ± 7.54	98.51 ± 6.44 b	207.43 ± 23.15	342.72 ± 26.10 b
*t*	0.012	2.613	0.129	5.191	0.020	3.917	0.192	5.667
*P*	0.990	0.010	0.898	0.001	0.984	0.001	0.848	0.001

The control group before and after treatment, *aP* < 0.05; the study group before and after treatment, *bP* < 0.05.

**Table 5:** Comparison of stress response indexes between the two groups before and after the operation ($\bar{x}$ ± *s*, *n* = 50).

Group	Cortisol (ng/ml)		Blood sugar (mmol/L)		CRP (mg/L)	
	Before operation	After operation	Before operation	After operation	Before operation	After operation
C	93.41 ± 20.05	127.44 ± 23.30a	4.55 ± 1.58	8.55 ± 1.37a	26.46 ± 8.31	62.58 ± 14.11a
R	93.38 ± 20.11	111.82 ± 16.29b	4.43 ± 1.15	7.72 ± 1.10b	26.50 ± 8.42	53.37 ± 11.85b
*t*	0.007	3.885	0.434	3.340	0.024	5.568
*P*	0.994	0.001	0.665	0.001	0.981	0.001

The control group before and after the treatment, *aP* < 0.05; the study group before and after the treatment, *bP* < 0.05.

## Data Availability

The datasets used and analyzed during the current study are available from the corresponding author upon reasonable request.
